# Case Report: Neurally adjusted ventilatory assist as an effective rescue treatment for pulmonary interstitial emphysema in extremely low birth weight infants

**DOI:** 10.3389/fped.2024.1332332

**Published:** 2024-01-22

**Authors:** Chien-Ming Chen, Mei-Yung Chung, Hong-Ya Kang, Mei-Chen Ou-Yang, Teh-Ming Wang, Chung-Ting Hsu

**Affiliations:** ^1^ Section of Neonatology, Department of Pediatrics, Kaohsiung Chang Gung Memorial Hospital and Chang Gung University College of Medicine, Kaohsiung, Taiwan; ^2^ Department of Respiratory Care, Kaohsiung Chang Gung Memorial Hospital, Kaohsiung, Taiwan; ^3^ Chang Gung University of Science and Technology, Chiayi Campus, Chiayi, Taiwan; ^4^ Children’s Medical Center, Taichung Veterans General Hospital, Taichung, Taiwan; ^5^ Department of Post-Baccalaureate Medicine, College of Medicine, National Chung Hsing University, Taichung, Taiwan; ^6^ Department of Biomedical Engineering & Environmental Sciences, National Tsing Hua University, Hsinchu, Taiwan

**Keywords:** neurally adjusted ventilatory assist, pulmonary interstitial emphysema, extremely low birth weight infants, non-invasive neurally adjusted ventilatory assist, case report

## Abstract

Pulmonary interstitial emphysema (PIE) is a complication observed in extremely low birth weight (ELBW) infants on mechanical ventilation. Despite various proposed therapeutic interventions, the success rates have shown inconsistency. Neurally adjusted ventilatory assist (NAVA) stands out as a novel respiratory support mode, offering lower pressure and tidal volume in comparison to conventional ventilation methods. In this case report, we present five ELBW infants with refractory PIE who were transitioned to NAVA ventilation. Following the switch to NAVA, all cases of PIE gradually resolved. In contrast to traditional modes, NAVA provided respiratory support with significantly lower fraction of inspired oxygen, reduced peak inspiratory pressure, diminished mean airway pressure, and decreased tidal volume within 7 days of NAVA utilization (*p* = 0.042, 0.043, 0.043, and 0.042, respectively). Consequently, we propose that NAVA could serve as a valuable rescue treatment for ELBW infants with PIE.

## 1 Introduction

Despite the advances in neonatology, such as the administration of prenatal steroids, postnatal surfactant, and the implementation of gentle ventilation, the increased survival of premature infants is still accompanied by pulmonary morbidities, including bronchopulmonary dysplasia (BPD), pulmonary hypertension, and pulmonary interstitial emphysema (PIE), especially in extremely low birth weight (ELBW) infants (1, 2). PIE is characterized by the presence of air leaks in interstitial or perivascular lung tissue entrapped along bronchovascular bundles, leading to hypoxemia and respiratory acidosis (3). Ventilator settings are frequently increased due to impaired gas exchange, potentially aggravating air trapping and resulting in further deterioration of ventilation and oxygenation (4, 5). The management of PIE can be challenging, and previous studies have proposed various treatment modalities with varying degrees of success, such as the lateral decubitus position, gentle ventilation with reduced inspiratory time, decreased peak inspiratory pressure (PIP), adjusted positive end-expiratory pressure (PEEP), high-frequency oscillatory ventilation (HFOV), selective main bronchial intubation or occlusion, lung puncture, and even lobectomy (6–8).

In recent years, neurally adjusted ventilatory assist (NAVA) has been increasingly used in neonatal intensive care units (NICUs) (9). Theoretically, NAVA holds the potential to improve PIE by delivering lower PIP and lower work of breathing compared to synchronized intermittent mandatory ventilation (SIMV) (10, 11). However, there are limited studies on the use of NAVA for premature infants with PIE (12, 13). In this report, we present five extremely low birth weight premature infants with PIE who were unresponsive to conventional treatments but demonstrated significant improvement after being ventilated with NAVA.

## 2 Case description

Five ELBW infants were included in this case report. Out of these cases, three were diagnosed with localized PIE, while the remaining two had diffuse PIE. The conventional PIE management, including lateral decubitus position, gentle ventilation by reducing inspiratory time, decreasing PIP, adjusting PEEP, and transitioning to HFOV or non-invasive ventilation mode, was applied initially. Unfortunately, these traditional treatment methods for PIE proved ineffective in all cases, leading to the implementation of NAVA.

### 2.1 NAVA initial settings

The decision to switch to NAVA is based on the following specific criteria: First, the absence of improvement or worsening in PIE on chest x-ray after at least 48 h of attempting traditional ventilation adjustments. Second, clinical observation of increased episodes of bradycardia and cyanosis.

After transitioning to NAVA mode, the initial step involved determining the Breakpoint, which refers to the appropriate NAVA level for the initial setting (14, 15). This process commenced at a NAVA level of 0.5 cmH2O/µV and involved gradually increasing the level by 0.5 cmH2O/µV every 30–60 s of continuous monitoring. The adjustment persisted until the PIP reached a plateau, and the electrical activity of the diaphragm (Edi) peak level displayed a consistent downward trend, indicating the establishment of the Breakpoint. Notably, previous studies have not suggested a NAVA level exceeding 4 cmH2O/µV in neonates (16, 17).

The Edi trigger was recommended to be set at 0.5 µV. Additionally, the alarm pressure limit was set to be 5 cmH2O above the PIP reached during the patient's spontaneous breathing (9). To maintain oxygen saturation (SpO2) within the target range of 90%–95%, the fraction of inspired oxygen (FiO2) was adjusted accordingly. Given the high risk of apnea in ELBW infants, a short apnea time from 2 s was suggested. Furthermore, the backup ventilation settings were mirrored from the previous mode before transitioning to NAVA.

### 2.2 Strategy of NAVA adjustment

The patient was evaluated twice per day. If the patient's Edi peak level was consistently below 10 µV and vital signs remained stable, we would gradually taper the NAVA level by 0.1–0.2 cmH2O/µV. Additionally, we would gradually increase apnea time and decrease backup mode settings during episodes of fewer bradycardia and cyanosis clinically observed. Extubation would be considered if the NAVA level was maintained below 0.8–1.0 cmH2O/µV, and apnea time could be safely increased to 5 s.

After the extubation process, the patient transitioned to non-invasive NAVA (NIV NAVA). We increased the NAVA level by 0.5–1.0 cmH2O/µV post-extubation. Simultaneously, the apnea duration was adjusted back to 2 s. Using the same approach, we gradually reduced the NAVA level by 0.1–0.2 cmH2O/µV. Subsequently, the patient would be shifted to nasal continuous positive airway pressure (NCPAP) as soon as the NAVA level reached 0 cmH2O/µV, ensuring the patient achieves a clinically stable condition.

### 2.3 Clinical course in five cases

#### 2.3.1 Case 1

**Figure 1:**
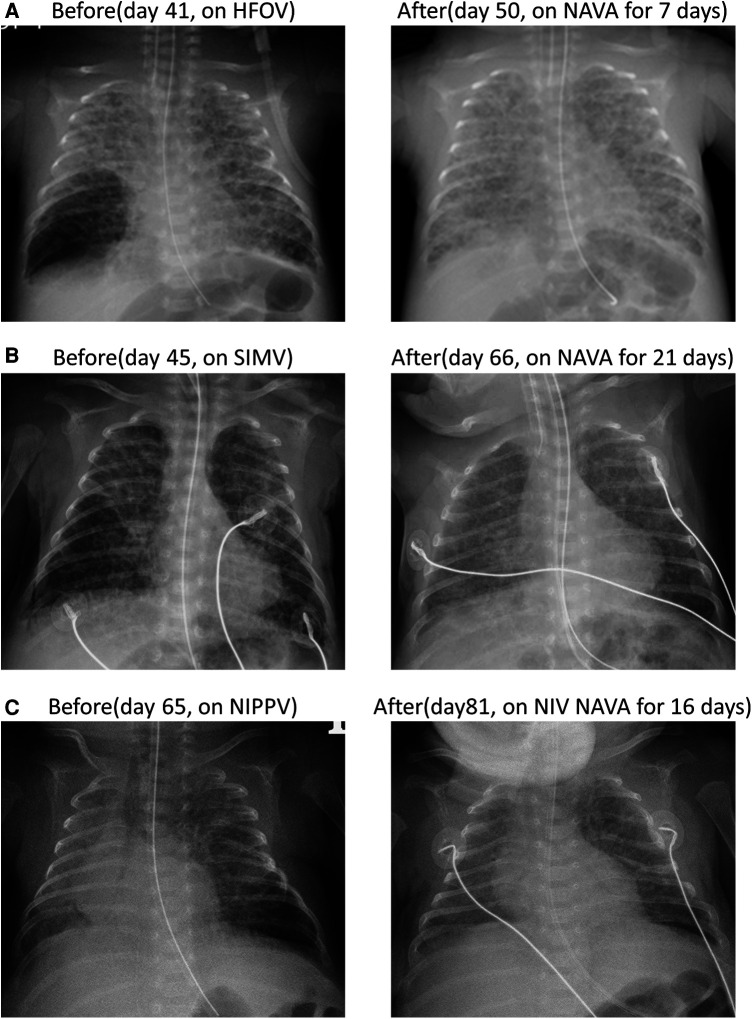
Three cases of localized PIE presented by anterior–posterior chest x-ray before and after NAVA. (**A**) In case 1, localized PIE over the right lower lobe was observed on day 43 and resolved on day 50 after 7 days of NAVA treatment. (**B**) In case 2, localized PIE over the right lower lobe presented on day 45 and resolved on day 66 after NAVA treatment for 21 days. (**C**) In case 5, localized PIE over left lower lobe on day 65 and completely resolved on day 81 after 16 days of NIV NAVA treatment.

A female premature neonate weighing 0.80 kg was delivered spontaneously at a gestational age (GA) of 24 + 6 weeks. Two doses of betamethasone were administered prenatally at a GA of 23 + 5 weeks. Following delivery, the neonate was subjected to nasal intermittent positive pressure ventilation (NIPPV). Due to an increasing FiO2, the neonate required intubation, and early rescue surfactant was administered twice. However, despite these interventions, the neonate experienced difficulty weaning from conventional synchronized ventilation. The patient was switched to HFOV with the aim of decreasing mean airway pressure (MAP) and reducing the risk of barotrauma after frequent apnea episode and an early type of bronchopulmonary dysplasia. However, we observed localized PIE in the right lower lobe on day 41, and the patient was subsequently positioned on the right lateral decubitus. On day 43, NAVA was initiated due to poor response to the right lateral decubitus position, along with the occurrence of bradycardia and cyanosis under HFOV at a reduced MAP setting. After 4 days of follow-up, we still noticed slight progression of PIE in the same area, accompanied by increased tidal volume. Nevertheless, the PIE situation significantly improved and stabilized after 7 days of NAVA usage, as evident from the chest x-ray, which showed complete resolution of PIE (shown in Figure 1A). Gradual weaning off NAVA was successful, and the neonate was extubated on day 91. After receiving NIV NAVA and NCPAP support, the patient did not require ventilator assistance until reaching a corrected age of 1.5 months.

#### 2.3.2 Case 2

A male premature infant with a GA of 25 + 5 weeks and a birth weight of 975 g was delivered via emergency cesarean section due to placental abruption. Prenatal steroids with 2 doses of betamethasone were administered at a GA of 24 weeks. Immediately after delivery, intubation was performed, and early rescue surfactant was administered once. Ventilator support was provided with the synchronized intermittent mandatory ventilation (SIMV) mode. The infant suffered from patent ductus arteriosus (PDA) ligation and recurrent pneumonia resulting in the collapse of multiple lobes. On day 42, PIE manifested in the right lower lobe. In response, we implemented the right lateral decubitus position. Additionally, we reduced PIP and shortened inspiratory time in SIMV mode. Despite these adjustments, frequent desaturation persisted. Consequently, NAVA was introduced on day 45 to address the evolving condition. After the switch to NAVA mode, the FiO2 could be gradually decreased. On day 66, the chest x-ray revealed complete resolution of the localized PIE (shown in Figure 1B). The infant was successfully extubated on day 67, but NIV NAVA and NCPAP were applied for an extended period due to BPD. At a corrected age of 3 months, the infant no longer required ventilator support.

#### 2.3.3 Case 3

**Figure 2:**
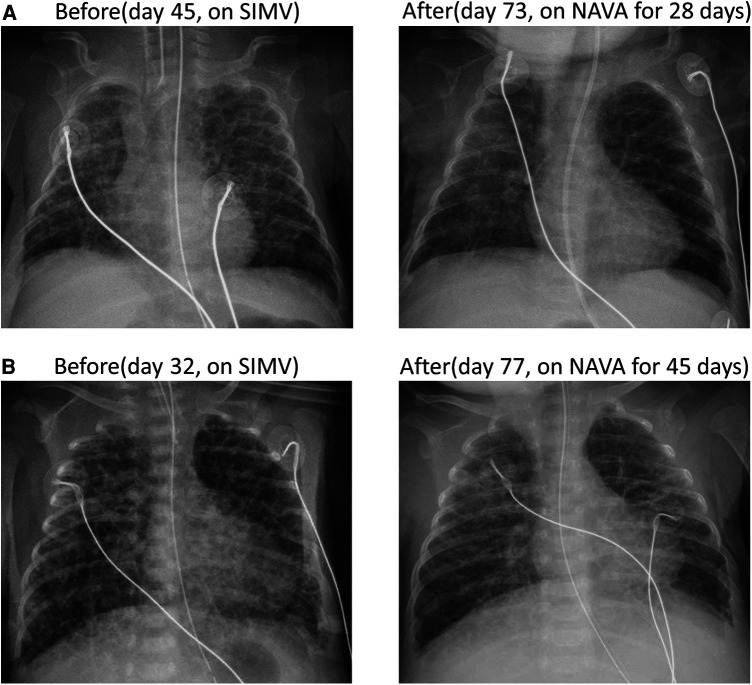
Two cases of diffuse PIE were observed through anterior-posterior chest x-ray before and after NAVA. (**A**) In case 3, diffuse PIE was observed on day 45 and resolved on day 73 after 28 days of NAVA and NIV NAVA treatment. (**B**) In case 4, diffuse PIE was detected on day 32 and resolved on day 77 after 45 days of NAVA and NIV NAVA treatment.

A female premature infant was delivered spontaneously at a GA of 24 + 1 weeks with a birth weight of 630 g. The mother received two doses of betamethasone as prenatal steroids 48 h before delivery. The infant was intubated and received surfactant administration due to respiratory distress syndrome (RDS), grade III. The PDA closed spontaneously on day 4. However, septic shock and bilateral intraventricular hemorrhage were noted on day 6, with both blood culture and placenta culture yielding E. coli. The infant developed obstructive hydrocephalus with increased intracranial pressure, requiring external ventricular drainage inserted by a neurosurgeon. On day 26, the infant developed diffuse PIE, and attempts were made to decrease PIP and shorten inspiratory time under SIMV mode to mitigate barotrauma. However, recurrent lung collapse with unstable saturation occurred frequently. On day 45, NAVA was initiated, leading to an improvement in PIE one day after the start of treatment. Additionally, the FiO2 was gradually decreased after using NAVA. The infant was extubated and shifted to NIV NAVA on day 68. PIE completely resolved after starting NAVA for four weeks (shown in Figure 2A). NCPAP was applied since day 85 due to BPD and periventricular leukomalacia, requiring ventilator support for a prolonged period. Weaning from NCPAP and discontinuing oxygen support were achieved at a corrected age of 6 months.

#### 2.3.4 Case 4

A female infant was delivered via cesarean section due to malpresentation at a GA of 26 + 5 weeks with a birth weight of 920 g. Preterm premature rupture of membranes with oligohydramnios was noted for 2 weeks prior to delivery. Prenatal steroids with 2 doses of betamethasone were administered 10 days before delivery. The infant was intubated immediately after birth and received ventilator support with SIMV mode after admission to the NICU. However, diffuse PIE occurred early on day 2, which may be attributed to hypoplastic lungs, induced by oligohydramnios during the prenatal stage. Based on the principles of lung protection and reducing barotrauma, the patient was extubated and shifted to NIPPV support. The PDA closed spontaneously on day 3. In the following days, progressive abdominal distension and frequent desaturation developed as a result of continuous positive airway pressure belly syndrome. Consequently, re-intubation was performed on day 24 due to severe cyanosis and bradycardia. PIE deteriorated severely after intubation even under decreasing PIP and shortening Ti in SIMV mode. The ventilator was shifted to NAVA mode on day 32. The level of saturation became more stable after NAVA ventilation, and the FiO2 level could be gradually tapered. The infant was extubated and then placed on NIV NAVA on day 65. PIE resolved completely on day 77 (shown in Figure 2B). Due to BPD, the infant needed NCPAP support until corrected age of 3 months.

#### 2.3.5 Case 5

A male premature infant was spontaneously delivered at a GA of 24 + 2 weeks, weighing 640 g. Prenatal steroids, including two doses of Dexamethasone, were administered 24 h before delivery. Due to bradycardia and cyanosis, immediate intubation was performed after birth. Upon admission to the NICU, the infant was supported with a ventilator using SIMV mode. On day 8, the infant's right lung collapsed, and pneumonia was diagnosed, prompting a switch to HFOV ventilation mode. The infant underwent ligation of a hemodynamically significant PDA and received peritoneal drainage and broad-spectrum antibiotics for necrotizing enterocolitis with gastrointestinal perforation. The infant's respiratory condition gradually improved, leading to extubation on day 51 and use of NIPPV. However, on day 60, PIE was noted over the left lower lobe due to the high settings of NIPPV for abdominal distention. Despite our attempts to address the situation by placing the infant in the left lateral decubitus position and decreasing PIP and PEEP levels of NIPPV, frequent episodes of bradycardia and cyanosis persisted. Consequently, NIV NAVA was initiated on day 65. Following this intervention, oxygen saturation became notably more stable, and abdominal distention also improved. A series of chest x-rays revealed gradual improvement of PIE, which completely resolved on day 81 (shown in Figure 1C). At postmenstrual age of 41 + 2 weeks, the infant was able to tolerate room air without ventilator support.

### 2.4 NAVA effectiveness in PIE resolution

The clinical characteristics of these five ELBW infants were summarized in Table 1. All of them had received prenatal steroids before delivery and were diagnosed with RDS after birth. Among these cases, only three infants received early rescue surfactant treatment due to an increasing demand for oxygen during ventilation. The criterion for surfactant administration was that the patient required an FiO2 above 0.4 to maintain SpO2 above 90%. We all use traditional method for surfactant administration via endotracheal tube in ELBW infants. In the fifth case, PIE developed while the patient was undergoing non-invasive ventilation after extubation. Consequently, we utilized NIV NAVA directly.

**Table 1:** Demographic data of 5 cases.

	Case 1	Case 2	Case 3	Case 4	Case 5
Gestational age (weeks)	24	25	24	26	24
Birth weight (grams)	800	975	630	920	640
Gender	Female	Male	Female	Female	Male
Prenatal steroid	Twice	Twice	Twice	Twice	Twice
Surfactant use	Twice	Once	Once	None	None
Onset of PIE	41th day	42th day	26th day	2nd day	60th day
Type of PIE	Localized	Localized	Diffuse	Diffuse	Localized
Ventilator mode before NAVA	HFOV	PC-SIMV	PC-SIMV	PC-SIMV	NIPPV
Treatments before NAVA	Lateral decubitus MAP↓in HFOV	Lateral decubitus PIP↓, Ti↓in SIMV	PIP↓, Ti↓in SIMV	Non-invasive mode PIP↓, Ti↓in SIMV	Lateral decubitus PIP/PEEP↓in NIPPV
Timing of switching to NAVA	43th day	45th day	45th day	32th day	65th day
Initial NAVA level (cmH2O/µV)	1.5	1.5	2.5	2.5	2.5
Total duration of NAVA (Days)	59	38	40	60	26
NAVA	45	22	23	33	-
NIV NAVA	14	16	17	27	26
Duration of PIE resolution after NAVA (Days)	7	21	28	45	16

HFOV, high frequency oscillatory ventilation; MAP, mean airway pressure; NAVA, neurally adjusted ventilatory assist; NIPPV, nasal intermittent positive pressure ventilation; NIV NAVA, non-invasive neurally adjusted ventilatory assist; PC, pressure control; PEEP, positive end-expiratory pressure; PIE, pulmonary interstitial emphysema; PIP, peak inspiratory pressure; SIMV, synchronized intermittent mandatory ventilation; Ti, inspiratory time.

Chest x-rays were performed daily in the first 3 days after transitioning to NAVA, with the frequency subsequently decreasing to once every 2–3 days based on clinical decision until PIE resolution. The complete resolution of PIE is determined through radiological imaging and assessed by two or more clinical physicians. Based on our experience, we observed successful resolution of PIE in all five cases following NAVA treatment. The initial settings of NAVA level are around 1.5–2.5 cmH2O/µV after switching to NAVA mode. The localized PIE typically resolved within one to three weeks after NAVA treatment, whereas diffuse PIE required a longer period of time, often exceeding four weeks. Throughout the duration of NAVA treatment, no complications were observed. Although NAVA was effective in resolving PIE, all cases still developed BPD due to severe lung trauma. However, eventually all five of these cases were weaned off the ventilator and were able to tolerate room air without requiring oxygen support later in life.

### 2.5 Ventilator setting reduction following NAVA

**Figure 3:**
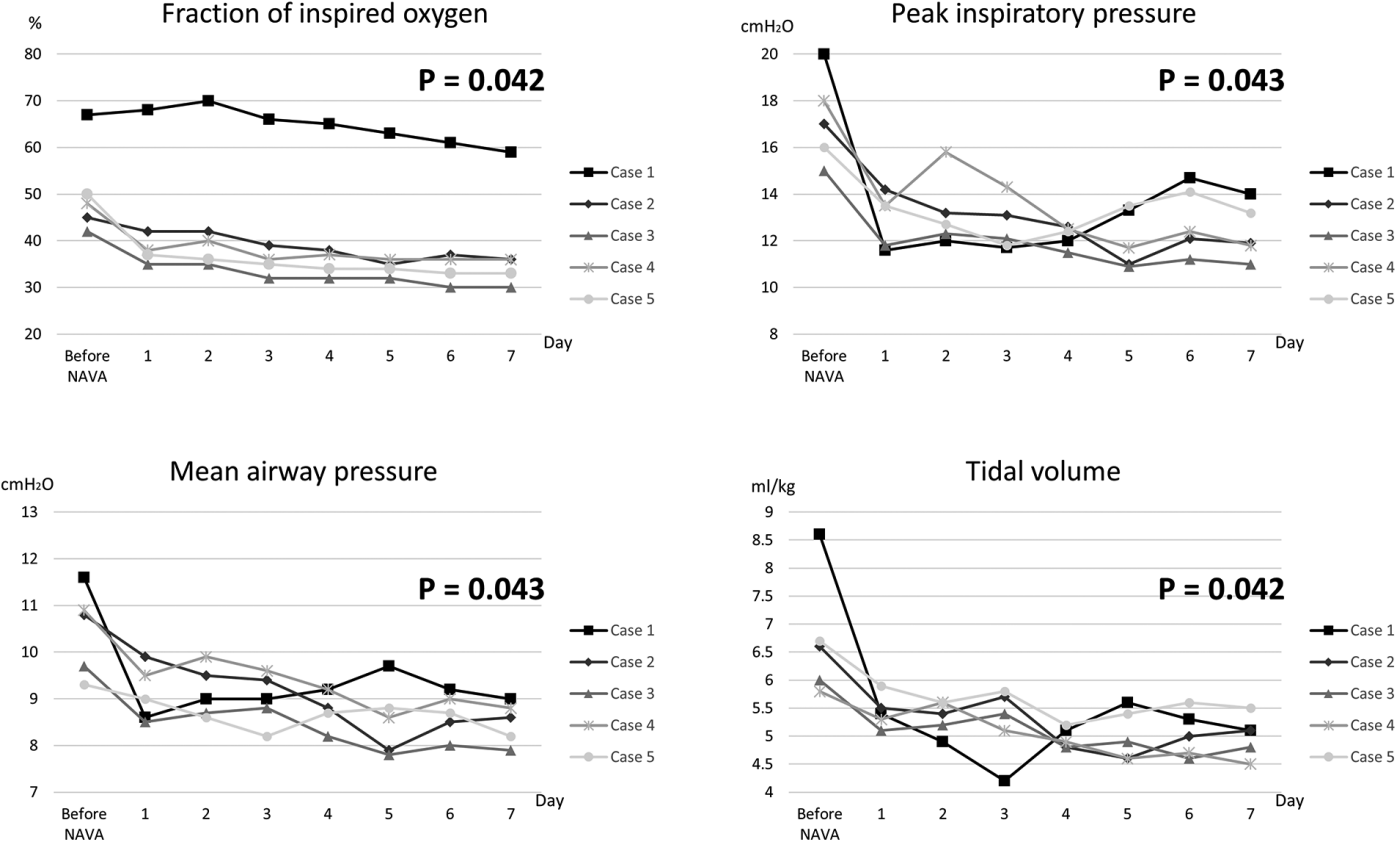
The daily ventilator settings and measurements for all five cases before and after the transition to NAVA over a duration of 1 week.

Figure 3 illustrates the daily ventilator settings and measurements before and one week after the transition to NAVA in all five cases. Following the switch to NAVA mode, a significant downward trend was observed in several parameters, including FiO2, PIP, MAP, and tidal volume, within the initial 7 days. The corresponding *p*-values for these changes were calculated by the Wilcoxon signed-rank test as 0.042, 0.043, 0.043, and 0.042, respectively. Clinically, it was confirmed that all five cases achieved respiratory stability following the shift to NAVA. Moreover, the frequency of bradycardia and cyanosis episodes was lower compared to the traditional ventilator modes.

## 3 Discussion

In our study, the intractable PIE in ELBW infants was successfully resolved following treatment with NAVA ventilation. NAVA has demonstrated substantial potential in reducing oxygen demand, peak inspiratory pressure, mean airway pressure, and tidal volume in ELBW infants compared to conventional ventilation methods. NAVA utilizes the patient's Edi to trigger and deliver synchronized and proportional assisted ventilation. The good synchronization achieved by NAVA also diminishes the necessity for sedation with narcotics (18, 19). Previous studies have indicated that both NAVA and NIV NAVA provide superior patient-ventilator interaction and gas exchange, resulting in reduced oxygen requirements, decreased peak inspiratory pressure, and diminished respiratory muscle load when compared to conventional ventilation methods (9, 20). These factors are believed to be the key reasons why NAVA shows promise in alleviating PIE.

In terms of determining the breakpoint in NAVA initial settings, two earlier studies advocate a gradual increase of 0.5 cmH2O/µV every 3 min (14, 15). However, an alternative recommendation was proposed in another review article, suggesting an increase every 30 s (9). Based on our clinical experience, we have found that a 30- to 60-second interval is adequate for observing a typical plateau, particularly when the patient is calm. We used a cutoff level of 10 µV for Edi peak to assess NAVA level adjustments. This choice was informed by previous observational studies, which indicated that the normal range of Edi peak in premature babies is approximately 10.8 ± 3.7 µV (21). Lee et al. (12) has also shown a downward trend in FiO2, PIP, and tidal volume after switching to NAVA mode for PIE management, mirroring the results of our study. This consistency suggests that the mechanism of PIE treatment with NAVA involves gentle ventilation and synchronization. However, in this previous case report (12), the initial NAVA level was set at 3 cmH2O/µV, which is higher than in our study. A letter to the editor (22) highlighted the concern about setting the NAVA level above 2.5 cmH2O/µV, suggesting it might provide excessive assistance to premature infants and lead to lower Edi peak levels, as observed in the aforementioned case report.

Traditionally, the radiological manifestation of PIE has been observed in ELBW infants with RDS who were ventilated with a mechanical ventilator (23). The mechanism of PIE is characterized by the hyperdistention of alveoli and terminal airways, ultimately leading to tissue rupture. Air leaks and alveolar rupture may occur due to various factors, such as mechanical ventilation, positive pressure ventilation, uneven ventilation, and reduced lung compliance, especially in cases of premature lungs that are highly sensitive to stretch. The increased transpulmonary pressure that exceeds the tensile strength of the alveoli and airways damages the respiratory epithelium, resulting in the entry of air into the interstitial tissue of the lung due to elevated intra-alveolar pressure (5, 6, 24, 25). Currently identified etiologies of PIE comprise prematurity, extremely low birth weight, RDS, positive pressure or mechanical ventilation employing high peak pressure, high tidal volume or prolonged inspiratory time, meconium aspiration syndrome, amniotic fluid aspiration, pulmonary infection, pulmonary hypoplasia, and improper positioning of the endotracheal tube (3, 6, 26, 27). All five of our ELBW premature infants were diagnosed with RDS and underwent intubation with mechanical ventilation. Pulmonary infection episodes were also noted prior to the development of PIE in cases 2 and 5. The cause of PIE in our study is readily apparent.

The fundamental treatment approach for PIE involves utilizing gentle ventilation techniques to decrease barotrauma and volutrauma (28). This includes non-invasive ventilation, volume control, or high-frequency ventilation, all of which have been found effective in minimizing the development of PIE (8, 29). Other treatment methods that have been reported to successfully resolve PIE include lateral decubitus position, selective main bronchial intubation or occlusion, lung puncture, and lobectomy (7, 30–36). In our case report, we attempted conventional strategies such as the lateral decubitus position, gentle ventilation with shortened inspiratory time, reduced PIP, adjusted PEEP, HFOV with decreased MAP, and non-invasive ventilation. Unfortunately, these measures were unsuccessful in resolving PIE. The interval between the onset of PIE and the initiation of NAVA varied among our five patients, ranging from 2 days–30 days. This considerable variability is attributed to parental hesitation regarding the substantial expense of NAVA treatment and individual differences in the clinical course.

Previous studies have shown that NAVA can provide the benefits of delivering optimal volume and precise cycling-off based on the patient's own Edi (37). This is advantageous in treating patients with PIE, particularly in addressing discrepancies between both lungs and preventing further overdistension of PIE or under-ventilation of the contralateral lung (13, 37). These advantages are not possible with traditional synchronized ventilation methods, as achieving a balanced tidal volume and accurate inspiratory time for both lungs without Edi is difficult for physicians. Compared to conventional synchronized ventilation, HFOV and non-invasive ventilation, NAVA appears to be a more effective treatment for patients with PIE. Additionally, NAVA is safer and less invasive than other treatments such as selective main bronchial intubation or occlusion, lung puncture, and lobectomy, and does not require sedation (13).

Before introducing NAVA for the treatment of PIE in our units, we were limited to traditional methods involving ventilator adjustments. This approach often resulted in lung collapse or the progression of PIE, complicating matters with severe BPD later. Such complications led to patient mortality or the necessity for home ventilator support after discharge.

Our study has certain limitations. NAVA is a relatively novel ventilation mode, and our clinical experience in manipulating NAVA is limited compared to traditional modes in ELBW infants. This comparative lack of experience could result in conservative management during the weaning process of NAVA, potentially prolonging the resolution period of PIE. Additionally, our study suffered from a small sample size, attributed to a data collection period of only two years across two medical centers. It is worth noting that NAVA is a self-pay treatment in Taiwan, and the high cost of the Edi catheter makes it unaffordable for many parents, contributing to the limitations in our sample size during collection. Consequently, further research with a larger sample size is imperative to validate our findings.

## 4 Conclusion

Based on our limited experience, we suggest that NAVA may be an effective rescue treatment option for patients with PIE. However, further larger studies are necessary to evaluate its effectiveness.

## Data Availability

The original contributions presented in the study are included in the article/Supplementary Material, further inquiries can be directed to the corresponding author.
